# Genetic Analyses in Dent Disease and Characterization of *CLCN5* Mutations in Kidney Biopsies

**DOI:** 10.3390/ijms21020516

**Published:** 2020-01-14

**Authors:** Lisa Gianesello, Monica Ceol, Loris Bertoldi, Liliana Terrin, Giovanna Priante, Luisa Murer, Licia Peruzzi, Mario Giordano, Fabio Paglialonga, Vincenzo Cantaluppi, Claudio Musetti, Giorgio Valle, Dorella Del Prete, Franca Anglani, Dent Disease Italian Network

**Affiliations:** 1 Laboratory of Histomorphology and Molecular Biology of the Kidney, Clinical Nephrology, Department of Medicine—DIMED, University of Padua, 35128 Padua, Italy; lisa.gianesello@unipd.it (L.G.); monica.ceol@unipd.it (M.C.); liliana.terrin@gmail.com (L.T.); giovanna.priante@unipd.it (G.P.); dorella.delprete@unipd.it (D.D.P.); 2 CRIBI Biotechnology Centre, University of Padua, 35131 Padua, Italy; loris.bertoldi@phd.unipd.it (L.B.); giorgio.valle@unipd.it (G.V.); 3 Pediatric Nephrology, Dialysis and Transplant Unit, Department of Women’s and Children’s Health, Padua University Hospital, 35128 Padua, Italy; luisa.murer@aopd.veneto.it; 4 Pediatric Nephrology Unit, Regina Margherita Children’s Hospital, 10126 CDSS Turin, Italy; licia.peruzzi@unito.it; 5 Pediatric Nephrology Unit, University Hospital, P.O. Giovanni XXIII, 70126 Bari, Italy; mario.giordano@policlinico.ba.it; 6 Pediatric Nephrology, Dialysis and Transplant Unit, Fondazione IRCCS, Ca’ Granda Ospedale Maggiore Policlinico, 20122 Milan, Italy; fabio.paglialonga@policlinico.mi.it; 7 Nephrology and Kidney Transplantation Unit, Department of Translational Medicine, University of Piemonte Orientale (UPO), 28100 Novara, Italy; vincenzo.cantaluppi@med.uniupo.it (V.C.); claudio.musetti@med.uniupo.it (C.M.)

**Keywords:** dent disease, *CLCN5* gene mutations, proximal tubular ClC-5 expression, megalin, cubilin, kidney biopsies, immunohistochemistry, whole exome sequencing

## Abstract

Dent disease (DD), an X-linked renal tubulopathy, is mainly caused by loss-of-function mutations in *CLCN5* (DD1) and *OCRL* genes. *CLCN5* encodes the ClC-5 antiporter that in proximal tubules (PT) participates in the receptor-mediated endocytosis of low molecular weight proteins. Few studies have analyzed the PT expression of ClC-5 and of megalin and cubilin receptors in DD1 kidney biopsies. About 25% of DD cases lack mutations in either *CLCN5* or *OCRL* genes (DD3), and no other disease genes have been discovered so far. Sanger sequencing was used for *CLCN5* gene analysis in 158 unrelated males clinically suspected of having DD. The tubular expression of ClC-5, megalin, and cubilin was assessed by immunolabeling in 10 DD1 kidney biopsies. Whole exome sequencing (WES) was performed in eight DD3 patients. Twenty-three novel *CLCN5* mutations were identified. ClC-5, megalin, and cubilin were significantly lower in DD1 than in control biopsies. The tubular expression of ClC-5 when detected was irrespective of the type of mutation. In four DD3 patients, WES revealed 12 potentially pathogenic variants in three novel genes (*SLC17A1*, *SLC9A3*, and *PDZK1*), and in three genes known to be associated with monogenic forms of renal proximal tubulopathies (*SLC3A*, *LRP2*, and *CUBN*). The supposed third Dent disease-causing gene was not discovered.

## 1. Introduction

The term Dent disease (DD) identifies a group of X-linked renal disorders characterized by features of incomplete Fanconi syndrome including low-molecular-weight proteinuria (LMWP), and more or less severe hypercalciuria, nephrocalcinosis and/or nephrolithiasis. This triad of symptoms has been variously named in the past as X-linked recessive nephrolithiasis with renal failure (OMIM 310468), X-linked recessive hypophosphatemic rickets (OMIM 300554), or the idiopathic LMWP of Japanese children (OMIM 308990), testifying to the disease’s phenotypic variability [1,2]. DD usually presents in children or young adults, progressing to chronic kidney disease (CKD) between the third and fifth decades of life in 30–80% of cases [3,4].

The most common genetic cause of DD is a mutated *CLCN5* gene encoding the ClC-5 chloride channel Cl-/H+ antiporter (DD1; MIM#300009) [5,6,7,8,9]. In the kidney, ClC-5 is expressed primarily in the proximal tubular cells (PTCs) located mainly in the subapical endosomes. Together with megalin and cubilin synergistic receptors, it is involved in the endocytic reabsorption of albumin and LMW proteins [10,11]. ClC-5 expression levels are lower in the α intercalated cells of the cortical collecting duct and in the cortical and medullary thick ascending limb of Henle’s loop [12].

DD1 features a marked allelic heterogeneity, with more than 200 *CLCN5* mutations described so far [9]. Functional investigations in Xenopus Levis oocytes and mammalian cells enabled these *CLCN5* mutations to be classified. The most common mutations lead to a defective protein folding and processing, resulting in endoplasmic reticulum (ER) retention of the mutant protein for further degradation by the proteasome [13,14,15,16,17]. Few studies have investigated ClC-5 expression in DD1 kidney biopsies.

*OCRL* gene mutations, which are usually associated with Lowe syndrome (OMIM #309000), have been identified in about 10–15% of DD patients (DD2; MIM#300555). Approximately 25% of DD patients (DD3) have neither *CLCN5* nor *OCRL* gene mutations [18,19,20,21].

This study aimed to investigate allelic and locus heterogeneity in DD and to analyze ClC-5, megalin, and cubilin expression in DD1 kidney biopsies. We further expanded the spectrum of *CLCN5* mutations in DD by describing 23 novel mutations. In DD1 kidney biopsies, we showed that the loss of ClC-5 tubular expression caused defective megalin and cubilin trafficking. In DD3, whole exome sequencing (WES) did not detect a new disease-causing gene. Instead, it revealed the concomitant presence of likely pathogenic variants in genes encoding proximal tubular (PT) endocytic apparatus components, suggesting that they may have had a role in determining the DD3 phenotype.

## 2. Results

### 2.1. CLCN5 Gene Mutation Analysis

**Figure 1:**
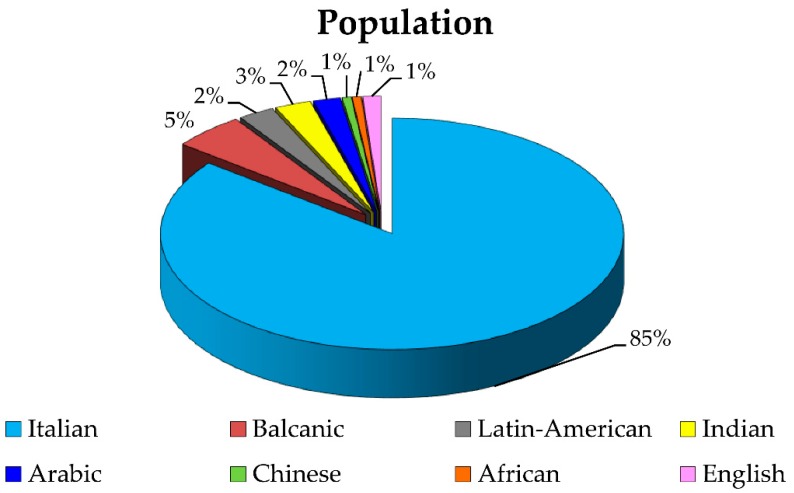
Ethnical distribution of the 158 analyzed patients.

The 85% of the 158 patients analyzed for the presence of *CLCN5* mutations were of Italian origin, 6% were non-Italian European (Balcanic and English), and the remaining 9% were extra-European (Figure 1).

**Figure 2:**
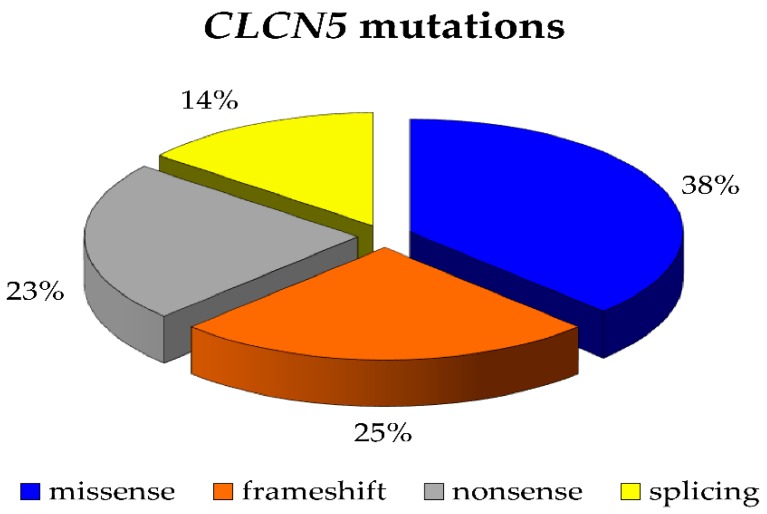
Percentages of mutations of *CLCN5* gene by type.

DNA sequence analysis of the *CLCN5* gene revealed 50 different mutations in 56 unrelated patients. Six different mutations were found twice. Among the detected mutations, the most common types were missense mutations (21 cases), followed by frameshift mutations (14 cases), nonsense mutations (13 cases), and splicing mutations (eight cases) (Figure 2).

Twenty-three mutations were not previously described, which were judged potentially pathogenic by in silico tools and classified as pathogenic or likely pathogenic according to American College of Medical Genetics and American College of Pathologists (ACMG/AMP) guidelines [22] (Table 1). The novel frameshift, nonsense, and missense mutations were mapped onto ClC-5 protein domains (Table 1). Table S1 summarizes the clinical details of 20 patients with novel *CLCN5* mutations (clinical data were unavailable for three). LMWP and hypercalciuria were the most common signs at the time of their molecular diagnosis, and their clinical phenotypic variability reflected that of patients with known *CLCN5* mutations [9].

### 2.2. ClC-5, Megalin, and Cubilin Immunolabeling in DD1 Kidney Biopsies

**Figure 3:**
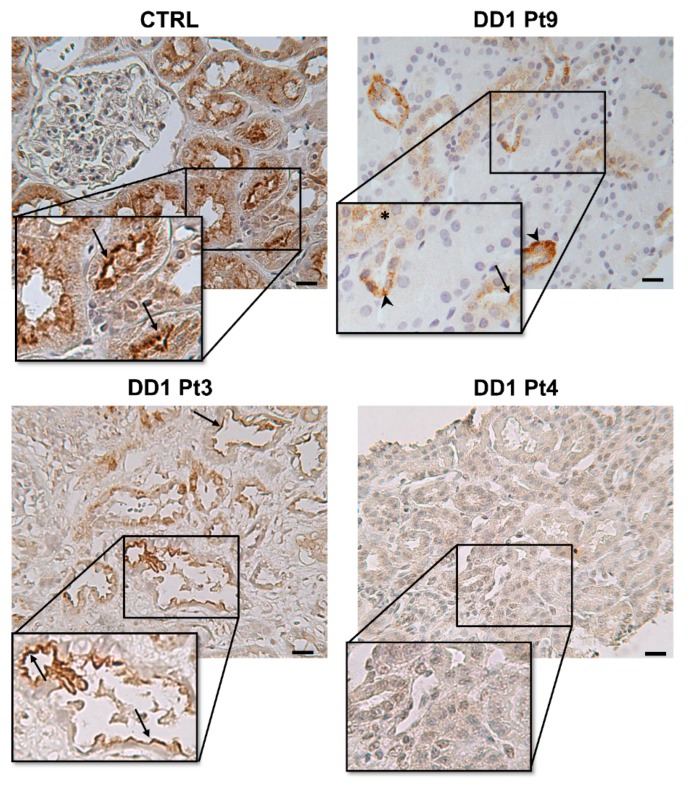
ClC-5 immunolabeling in control and DD1 kidneys. Representative images disclosing ClC-5 positivity in control and DD1 kidneys. In CTRL, ClC-5 staining was located mainly in tubular apical and subapical positions. In DD1 patients, some tubules presented basolateral or cytoplasmic ClC-5 positivity (Pt9), and very few showed apical staining (Pt9, Pt3). ClC-5 immunostaining was negligible in most DD1 tubules (Pt4), whatever the type of mutation. The asterisk indicates a cytoplasmic signal, arrows indicate apical and subapical signals, the arrowhead indicates a basolateral signal. Scale bar = 50 µm. CTRL= control, Pt = patient.

Renal tubular ClC-5 expression was analyzed by immunohistochemistry (IHC) in 10 patients carrying *CLCN5* stop codon (frameshift and nonsense mutations) or missense mutations (Table 2). In control biopsies, ClC-5 immunostaining was mainly apical and subapical (Figure 3). Our antibody has around 66% overall epitope sequence similarity to ClC-3 and ClC-4, which are both expressed in the membranes of intracellular organelles [23], so cross-reactivity can be expected. Immunolabeling for ClC-3, ClC-4, and ClC-5 in serial sections of a control sample showed that tubular apical staining was almost exclusively attributable to ClC-5 expression, while cytoplasmic staining was due largely to ClC-3 and ClC-5, and much less to ClC-4 (Figure S1).

**Figure 4:**
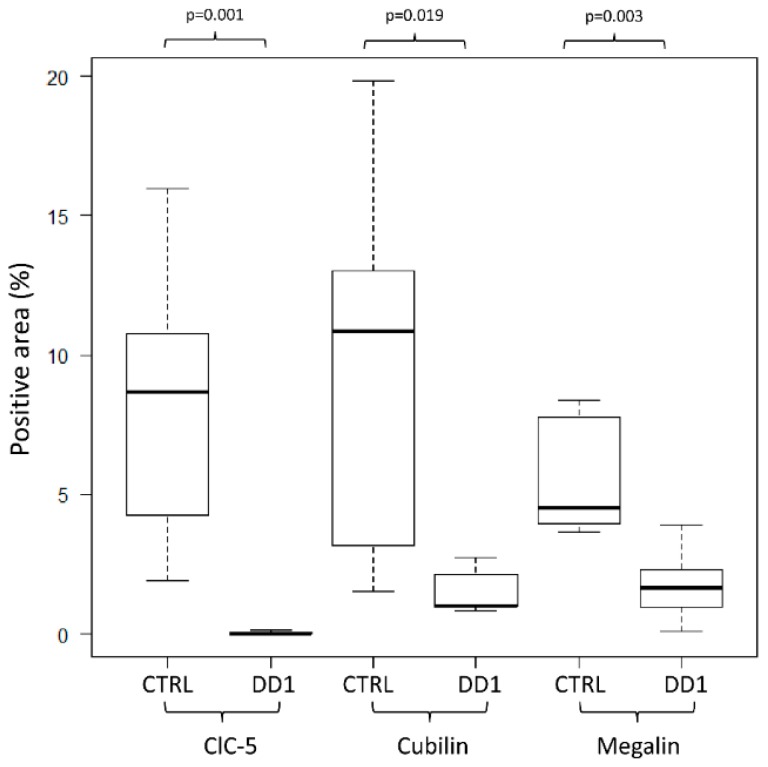
ClC-5, cubilin, and megalin quantitative analysis. Morphometric analysis showed a significant decrease in the percentage of positive area in kidneys of DD1 patients than in control kidneys for all molecules examined. Data were analyzed using the Mann–Whitney U-test. CTRL = control.

In DD1 biopsies, ClC-5 apical immunolabeling was negligible in most tubules, whatever the type of mutation (Figure 3). As expected, ClC-5 expression was very significantly downregulated (Figure 4) in DD1 biopsies compared with control biopsies (median CTRL 8.69% [Interquartile range (IQR) 1.94–15.97%], DD1 0.01% [IQR 0.00–0.12%]; *p* < 0.01).

Table 2 shows the morphometric findings on ClC-5 immunostaining for each mutation. Notably, two patients (Pt3 and Pt4) carried the same very premature nonsense mutation p.(Arg34*), but with a completely different pattern of expression: ClC-5 immunolabeling was completely absent in one, while in the other, it stained 0.12% of the whole biopsy area, which was more than in any of the other biopsies analyzed (Figure 3).

**Figure 5:**
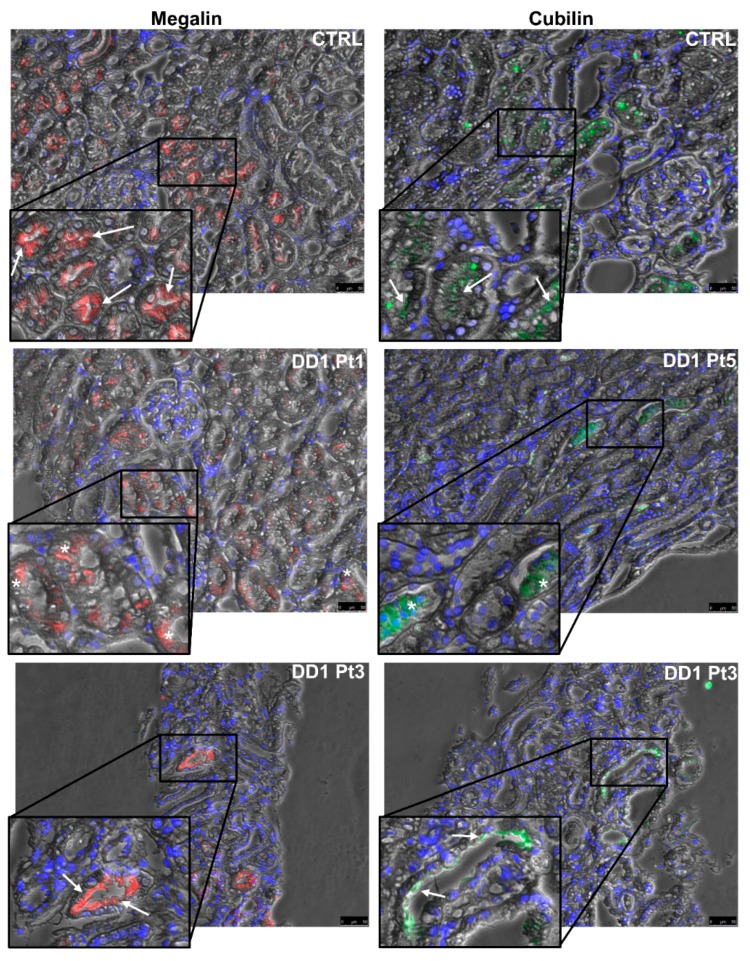
Megalin and cubilin immunolabeling in control and DD1 kidneys. Representative images disclosing megalin and cubilin positivity in control and DD1 kidneys. In CTRL, megalin (red) and cubilin (green) staining was located mainly in tubular apical and subapical positions. Immunolabeling for both receptors was rarely apical in DD1 patients (Pt3), while it was more frequently found in the cytoplasm (Pt1 and Pt5). The asterisk indicates a cytoplasmic signal, arrows indicate apical and subapical signals. Blue indicates counter-staining of nuclei with 4′,6-diamidino-2-phenylindole (DAPI). Scale bar = 50 µm. CTRL = control, Pt = patient.

Analyzing megalin and cubilin immunofluorescence (IF) in the same patients (Figure 5) revealed that both receptors were significantly downregulated by comparison with control biopsies (median megalin: CTRL 4.52% [IQR 3.64–8.39%], DD1 1.67% [IQR 0.09–3.91%], *p* = 0.019; median cubilin: CTRL 10.87% [IQR 1.54–19.85%], DD1 1.01% [IQR 0.83–2.67%], *p* = 0.003) (Figure 4).

### 2.3. Whole Exome Sequencing (WES) Study

Among *CLCN5* negative patients, 34 underwent mutational screening of the *OCRL* gene, and 19 patients were found to not carry mutations. In eight out of 19 *CLCN5* and *OCRL*-negative patients (four children and four adults), we performed WES.

We first searched for mutations in phenocopy genes. Known monogenic forms of nephrolithiasis and nephrocalcinosis as well as of proximal and distal tubulopathy, for a total of 62 genes including known genes of the PT endocytic pathway (Table S2), were firstly evaluated in these patients. Furthermore, the first 100 genes prioritized for their association with *CLCN5* or *OCRL* genes using the Scalable kernel-based gene prioritization (SCUBA) were investigated [24] (Table S3).

Unexpectedly, in two children, we detected *CLCN5* or *OCRL* known disease-causing mutations, p.(Lys231fs) and p.(Arg318Cys), respectively. If on one hand this finding was disturbing because it meant that by previous Sanger sequencing we had missed two causative mutations in the two known genes, on the other hand, it confirmed that our pool of DD3 cases was well representative of DD patients based on disease phenotype.

In four patients (three adults and one child), we detected 12 variants in three genes known to be associated either with monogenic forms of proximal renal tubulopathy (*SLC3A1*) or with monogenic syndromes involving proximal tubule dysfunction (*LRP2*, *CUBN*), as well as in novel genes not related to monogenic nephropathies (*SLC17A1*, *SLC9A3*, and *PDZK1*). These last genes could be candidates for DD-like phenotypes for their function in PT, and detected variants were predicted to be pathogenic or likely pathogenic by in silico tools (although with a different degree of concordance), except for one in the *SLC17A1* gene (Table 3). Table S4 summarizes the four patients’ clinical phenotypes.

In two adults patients (AMS and BDA), we detected biallelic likely pathogenic variants in *SLC3A1* and *LRP2* genes whose mutations are responsible for the recessive diseases Cystinuria (MIM#220100) and Donnai–Barrow/Facio-oculo-acoustico-renal syndrome (DB/FOAR, MIM#222448), respectively.

In AMS, we also identified a very rare missense variant classified as a variant of uncertain significance (VUS) in the *SLC17A1* gene encoding sodium/phosphate cotransporter 1 (NPT1), which occurs at the apical pole of PTCs [25] and participates in renal urate export [26,27]. The same patient was found to be homozygous for the very rare nonsense variant p.(Arg8*) in the *PDKZ1* gene. This gene encodes the Na(+)/H(+) exchange regulatory cofactor NHE-RF3, which is a PDZ domain-containing scaffolding protein and one of the key molecules of the urate transportsome [28,29].

The already known pathogenic *LRP2* mutation p.(Asp2054Asn) [30] was detected in AMV. In this patient, we also found an in-frame indel variant of the *CUBN* gene encoding for cubilin. *CUBN* gene mutations are known to cause Imerslund–Gräsbeck syndrome (IGS, MIM#261100), which is an autosomal recessive disorder involving selective intestinal vitamin B12 malabsorption and LMWP. The p.(Val2347del) variant in *CUBN* is very rare (TOPmed 0.0000001); Mutation Taster (MT) and PROVEAN predicted its pathogenicity, and it was classified as VUS according to ACMG/AMP guidelines [22].

Among eight different *LRP2* uncommon coding variants with a minor allele frequency (MAF) < 0.05 detected in our DD3 patients, four were identified in one patient (AMT) of which two were predicted to be pathogenic by in silico tools (Table 3). Similar to AMV, this patient carried an uncommon *CUBN* missense variant that was considered pathogenic by MT, PROVEAN, and DANN, but classified as benign according to ACMG/AMP guidelines. He also harbored a very rare variant in the *SLC9A3* gene, which was predicted as pathogenic by in silico tools, and classified as VUS. The *SLC9A3* gene encodes sodium/hydrogen exchanger 3 (NHE3), which is the main apical Na+/H+ exchanger in adult kidneys [31], and part of the macromolecular endocytic complex at the brush border of PTCs [32,33].

## 3. Discussion

Dent disease 1 is a worldwide disease, and this is further confirmed by our cohort of patients which included persons from all over the word. More than 220 *CLCN5* pathogenic mutations have been reported so far. Mutations were found scattered along all exons of the gene and in different protein domains [9,34,35,36,37,38,39,40,41,42,43,44,45]. Mansour-Hendili et al. [9] reported that the majority were missense and frameshift mutations (33.33% and 29.05% respectively) followed by nonsense mutations (17.52%), splicing mutations (12.39%), and large deletions (4.70%). In our cohort of patients, missense and frameshift mutations were also the most frequent, but with a lower proportion (38% and 25% respectively), while we observed more nonsense mutations compared to the previously reported data (23%).

In our study, DNA sequence analysis of the *CLCN5* gene revealed 50 different mutations, 23 of which have never been described before. ACMG/AMP guidelines classify the nine missense novel mutations in *CLCN5* as likely pathogenic. They were mapped onto ClC-5 protein domains (Table 1).

The p.(Ile173Lys) missense mutation is in the D helix, which is one of the four helixes (D, F, N, and R) brought together near the channel center to form the Cl-selectivity filter [46] and consequently believed to alter ClC-5 conductance.

The p.(His731Pro) missense mutation affects the ClC-5 carboxy-terminus cytoplasmic domain. All eukaryotic ClCs have a large cytoplasmic C-terminus containing a pair of cystathionine beta-synthase (CBS) domains. Several authors have shown that CBS domains are involved in regulating the activity of ClCs, including ClC-5 [47,48,49]. Mutations affecting the two CBS domains were reported correctly targeted to the plasma membrane and early endosomes, but with altered ClC-5 electrical activity [14]. The nonsense mutation p.(Arg718*) truncating the ClC-5 protein near the C-terminus reportedly results in ER retention, underscoring the importance of the C-terminus in passing protein quality control in ER [50]. These findings suggest that truncated ClC-5 proteins at the C-terminus could cause function loss through defective protein processing. However, three different truncated mutations at the C-terminus—p.(Tyr617*), p.(Arg648*), and p.(Arg704*)—targeted the cell surface (albeit only one with residual activity) [50], so we cannot say whether our stop codon mutations at the C-terminus of ClC-5 protein (Table 1) could exhibit residual activity targeting the plasma membrane.

The p.(Ser203Trp) missense mutation affects the E helix, whose role in ClC-5 function is still unclear. The nearby p.(Leu200Arg) mutation reportedly produced a loss of Cl- conductance [1], and the p.(Ser203Leu) was found to cause current failures due to ER retention [50]. Taken together, these findings indicate that the p.(Ser203Trp) mutation is probably pathogenic.

Six missense mutations map in the major helixes (B, H, I, O, P, and Q) involved in dimer interface formation [9,46], or in the intervening loops, suggesting an impaired physical contact between the two subunits that might disrupt proper pore configuration [50,51]. In addition, for the p.(Ala540Val), a pathogenic missense in the same position in a Dent family from New Zealand has already been reported [38], confirming the possible damaging role of the alteration of this residue. The p.(Ser270Asn) missense mutation maps in the loop between helixes H and I, near the “proton glutamate” (Glu 268), which is crucial to the Cl-/H+ transport function [9]. Since the p.(Ser270Arg) mutation was reportedly associated with chloride current abolition [52], we hypothesize a similar effect of this new mutation.

Very few studies investigated ClC-5 expression in kidney biopsies [10,53]. We analyzed ClC-5 protein expression in kidney biopsies from 10 patients carrying three novel and seven known *CLCN5* mutations and in eight control biopsies. In controls, ClC-5 immunolabeling was mainly apical and subapical in tubular cells, and it was not co-localized with ClC-3 or ClC-4 staining. The few studies on ClC-5 expression in human kidney reported similar staining findings [10,53], which are justified by the well-accepted ClC-5 localization in early and recycling endosomes and the plasma membrane [54,55].

Our study is the first to demonstrate the loss of ClC-5 protein expression in DD1 kidneys. However, an apical staining was detected in very few tubules in 7/10 DD1 biopsies, including those with the novel p.(Val308Met) and p.(Ser203Trp) mutations. Therefore, we speculate that the expression of these ClC-5 mutants is regulated post-translationally, and mutated proteins can very rarely reach the plasma membrane. This is consistent with previous findings in ClC-5 mutant models. Both missense and nonsense ClC-5 mutants could be either targeted to early endosomes or plasma membrane, but with a limited activity, or confined to the ER [50]. In fact, some DD1 tubules were only labeled at the basolateral pole (probably a sign of ER retention) (Figure 3 Pt 9).

Apical staining was unexpectedly detected for the very premature truncated ClC-5 protein at codon 34. Premature stop codons (PSCs) account for one in two *CLCN5* mutations, and cause three distinct molecular alterations: (1) the production of a truncated, usually non-functional, protein; (2) degradation of the transcripts containing PSCs via the nonsense-mediated decay (NMD) pathway; and (3) exon skipping due to alternative cryptic acceptor or donor sites being used in the exon encompassing the stop codon [56]. The first molecular change can be excluded, because our ClC-5 antibody recognized an epitope at the C-terminus of the protein. The second and third might apply because (1) NMD may occasionally be bypassed when translational read-through allows the decoding of stop codons as sense codons, thus enabling protein translation; (2) PSCs can also prompt exon skipping by altering exonic splicing enhancer (ESE) or exonic splicing silencer (ESS) motifs. PSCs have even been found to be statistically inclined to induce exon skipping more than other exon mutations [57]. If this is true of the p.(Arg34*) mutation, we can expect exon skipping to be in frame, thus enabling complete protein synthesis and allowing the mutated protein to be detected by immunolabeling. However, such explanations for this ClC-5 mutant protein’s presence in one biopsy should be considered with caution, as the ClC-5 protein was not found in most tubules, nor in another biopsy carrying the same mutation. This could mean that how PSCs are processed by cell transcriptional and translational apparatus might depend on the context (meaning the cell environment and/or the genomic context).

As in *Clcn5* knock-out (KO) animal models [58], ClC-5 loss in human kidney causes defective cubilin and megalin recycling, leading to LMWP. All the ClC-5 mutants studied here triggered both their defective expression at the brush border of PTCs and their downregulation. The presence of a megalin signal at the apical border of some tubules in the biopsy carrying the p.(Arg34*) mutation (Figure 5, Pt3) suggests a residual ClC-5 activity enabling a normal endocytic process and consequent megalin recycling.

Few studies have examined megalin and cubilin expression in DD1. Urinary megalin excretion was found to be significantly lower in DD1 patients than in normal individuals [59]. IHC on kidney biopsies from two patients carrying different *CLCN5* mutations revealed a defective megalin, cubilin, and Dab2 expression in PTCs [60,61]. Studies on megalin recycling in conditionally immortalized proximal tubular epithelial cell lines from three patients with *CLCN5* mutations showed defects in cell surface expression and internalization [62]. Our data definitively corroborate previous findings and suggest that a reduced intracellular megalin and cubilin synthesis may also contribute to their defective apical exposure.

Approximately one in four DD patients have no *CLCN5* or *OCRL* gene mutations. Whether mutations in a third, as yet unknown gene can cause DD3 remains to be seen, but—judging from our WES study on six DD3 patients—this seems unlikely. Instead, as we previously suggested [63], WES data point to DD3 patients having atypical phenotypes of known hereditary nephropathies or blended phenotypes. In fact, we identified in two patients (AMS and BDA) biallelic likely pathogenic variants in two genes (*SLC3A1*, *LRP2*) whose mutations are known to cause cystinuria and DB/FOAR. Our findings suggest that probably these patients were misdiagnosed as DD because of the presence renal Fanconi syndrome. Indeed, several disorders are caused by mutations of genes coding for components of the endolysosomal system in the PT. Besides *CLCN5* (DD1) and *OCRL* (Lowe syndrome and Dent disease type 2) genes, they include *LRP2* (DB/FOAR), *CUBN*, *AMN* (Imerslund–Gräsbeck syndrome), and *CTNS* (nephropathic cystinosis). Typically, these recessive disorders cause proximal tubular dysfunction and lead to inappropriate urinary loss of LMW proteins and solutes (e.g., phosphate, glucose, amino acids, urate), and they often lead to renal failure. The clinical entity of generalized proximal tubular dysfunction is referred to as renal Fanconi syndrome.

However, apart from BDA who was found to carry biallelic pathogenic variants in the *LRP2* gene and, for this reason, and after a careful clinical revaluation, was assessed to suffer from an atypical form of DB/FOAR syndrome [64], the other patient (AMS) did not suffer from cystinuria, despite carrying biallelic variants in the *SLC3A1* gene that were classified as likely pathogenic according to ACMG/AMP variant interpretation. Indeed, in this patient, the urinary level of cysteine was found to be normal even after repeated measurements. Two hypotheses may explain these findings: (1) the variants may be hypomorphic, thereby allowing a limited gene product activity, and (2) the two variants are in the same allele (complex allele), although their MAF was highly different, suggesting the absence of a linkage disequilibrium.

Instead, what appears relevant from this study is finding in three patients (comprising AMS) possible pathogenic variants in more than one gene connected in functional networks (*PDZK1*, *SLC17A1*, *CUBN*, *SLC3A9*, and *LRP2*), which we considered important for explaining patients’ phenotypes, thus suggesting digenic or oligogenic disorders.

The major finding of WES study is the discovery in AMS of a homozygous truncating mutation in the *PDZK1* gene. This is a new gene that has never been related before to human diseases, although it is one of the loci of strongest effect on serum urate level and gut [65,66,67]. NHE-RF3 encoded by *PDZK1* is a major scaffolder protein in the brush border of kidney PTCs [68], interacting through its PDZ domain with key molecules of urate transport, including NPT1 [29]. Furthermore, NHE-RF3 is one of the several proteins interacting with the type-2a sodium phosphate cotransporter (NaPi-2a), which is the major inorganic phosphate cotransporter of the PTCs [69]. Targeted disruption of the *Pdzk1* gene by homologous recombination in mice induced modulation of the expression of selective ion channels in the kidney, including NaPi-2a. The steady-state levels of NaPi-2a were found to be reduced under a phosphorus (Pi)-rich diet, and this was paralleled by higher urinary total and fractional Pi excretion [69]. In these KO mice, serum urate was not measured, nor were urate transporters investigated. However, urine and serum analysis did not reveal any significant difference between KO and wild-type mice except for a significant increase in the cholesterol levels [69]. Interestingly, in *Pdzk1* KO mice under a high-Pi diet, the PDZ scaffolding protein NHE-RF1 was increased at the bush border of proximal tubules. NHE-RF1 was demonstrated to localize with megalin in the brush border, because it bounds to its internal C-terminal PDZ binding motif [70]. It was also showed that NHE-RF1 silencing in PTCs increased megalin expression [70].

In the same patient, we also detected a very rare missense variant in the *SLC17A1* gene, which is classified as VUS due to its extreme rarity in the human population (data from gnomAD: 1 allele out of 250846). The *SLC17A1* gene is one of the loci associated with serum urate level and gout [71] and encodes NPT1, which is a Cl-dependent urate transport interacting with NHE-RF3 encoded by the *PDZK1* gene [26]. The clinical phenotype of patient AMS involves multiple tubular defects (particularly hyperphosphaturia, hypercalciuria, and severe hypouricemia), which might be consistent with a partial renal Fanconi syndrome and had led to a clinical suspicion of renal hypouricemia (MIM#220150 and 612076) and atypical DD. By WES, we excluded the presence of pathogenic variants in both *SLC22A12* and *SLC2A9* genes encoding URAT1 and GLUT9, respectively. It is tempting to speculate that the *PDZK1* and the *SLC17A1* gene variants might have had a role in determining AMS clinical phenotype for their direct interaction with urate and phosphate transport. Family studies will help clarify these aspects.

Since megalin and cubilin PTC expression is altered in DD1, it is conceivable that *LRP2* and/or *CUBN* mutations can cause or contribute to a DD-like nephropathy. WES results seem to support this hypothesis. In addition to BDA, who has already been described as carrying biallelic mutations in the *LRP2* gene [64], AMV was also found to carry a known pathogenic *LRP2* allele and a very rare inframe deletion in the *CUBN* gene. *CUBN* variants have recently been associated with proteinuria with no signs of IGS. It was shown that a homozygous frameshift mutation in exon 53 of *CUBN* (p.Ser2785fs) only caused proteinuria [72]. Moreover, a missense variant in exon 57 (p.Ile2984Val) was associated with albuminuria [73]. Mutations in the *CUBN* gene cause IGS apparently only when they affect the cubilin–amnionless interaction domain (exons 1–20) or the IF-Cbl binding site (exons 21–29) [74]. In our patient, *CUBN* mutation is localized in exon 46. Follow-up showed that our patient’s LMWP was intermittent while his proteinuria started at 1 year old and ranged between 0.4 and 1 g/24 h.

True digenic inheritance (DI) is the simplest form for oligogenic disease [75], but it is also encountered when pathogenic mutations responsible for two different diseases are co-inherited, leading to a blended phenotype [76]. The two heterozygous mutations in the *LRP2* and *CUBN* genes, encoding proteins working close together on the same endocytic pathway, might plausibly be responsible for patient AMV’s disease phenotype. Further studies on his kidney biopsy and/or urinary proteoma might confirm this hypothesis. Family studies may help to solve these questions.

We detected two *LRP2* coding variants associated with two likely pathogenic missense variants in the *CUBN* and *SLC9A3* genes in the genome of a single patient (AMT). It is noteworthy that these genes respectively encode megalin, cubilin, and NHE3, which are located—together with ClC-5, amnionless, and Dab2—at the cell surface of PTCs, forming its endocytic apparatus [54,55].

*SLC9A3* homozygous or compound heterozygous disease-causing mutations have recently been reported in nine patients from eight families with congenital secretory sodium diarrhea (MIM#616868) [77]. No association has been found as yet between *SLC9A3* variants and renal proximal tubulopathies, but a defective Nhe3 exposure was found in *Clcn5* KO mice [55,78]. Studies by Gekle et al. [33] support a crucial role for NHE3 in proximal tubular receptor-mediated endocytosis by demonstrating in Nhe3 KO mice that Nhe3 deficiency led to a reduced protein reabsorption: the urinary protein patterns resembled those of mice deficient in megalin or ClC-5. Recent evidence also highlights the importance of NHE3 for calcium reabsorption. Nhe3 KO mice revealed significant urinary calcium wasting and a low cortical bone mineral density and trabecular bone mass [79].

The genetic data of the AMT patient are puzzling and raise some questions. With the advent of high-throughput sequencing, we are bound to discover more patients suffering from oligogenic diseases and learn more about how complex interactions between allelic and locus heterogeneity affect disease phenotypes [80]. The presence of multiple coding variants in the same gene, either in cis or in trans, may also conceivably cause defective protein functioning, although this needs to be demonstrated in animal and in vitro models [81]. The genotype–phenotype correlation in the AMT patient is worth investigating, because it seems to reveal such an impact on disease phenotype. This patient was 26 years old when referred to a nephrologist for kidney stones. His height (158 cm) and weight (42 kg) were below the third percentile. His renal phenotype mainly featured proximal tubulopathy manifesting as Fanconi syndrome with LMWP, hypercalciuria, hyperphosphaturia, glycosuria, natriuresis, and hypercitraturia. Therefore, the patient’s presenting phenotype was mainly related to an impaired renal calcium and phosphate metabolism. WES detected no relevant variants in genes known to be responsible for monogenic forms of nephrolithiasis and renal tubulopathies. We hypothesized that the AMT patient’s phenotype was due to the variants in *LRP2*, *CUBN*, and *SLC9A3*, whose products work on the same cellular pathways as ClC-5, or pathways related thereto, but this hypothesis needs to be evaluated by in vitro studies. From the point of view of the referring clinicians, tubular abnormalities might be a real challenge: indeed, clinical characteristics of different diseases sometimes overlap, and a full-blown classical phenotype (in the case of AMS, a renal Fanconi syndrome) is rare. Therefore, when multiple tubular defects (i.e., alteration in tubular handling of 2–4 different solutes) coexist, the chance of a blended phenotype becomes more plausible, and a next-generation sequencing (NGS) approach might be a good strategy to identify multiple (and possibly interacting) genetic defects that may explain each individual phenotype.

## 4. Material and Methods

### 4.1. Patients

#### 4.1.1. DNA Samples

From 2006 to 2018, DNA samples were collected from 158 unrelated pediatric and adult males with clinically suspected DD according to the criteria described in our previous study [82]. Patients should have encountered at least two of the above-mentioned criteria for being referred to our laboratory for a molecular diagnosis. Since proteinuria was recently reported as one of the DD symptoms in concomitance with signs of incomplete Fanconi tubulopathy [83], and because of the cost of urinary assessment of LMW proteins, we decided to include in the diagnostic workflow also patients presenting with proteinuria, although in the absence of documented LMWP. Informed consent to the genetic study was obtained from all probands or their parents.

DNA samples from eight patients (four children and four adults) with no detectable *CLCN5* or *OCRL* gene mutations underwent WES. The selection was based on the presence of a likely DD phenotype (i.e., the presence of LMWP and/or proteinuria, hypercalciuria, and at least one of the following: nephrocalcinosis, kidney stones, hypophosphataemia, renal failure, aminoaciduria, rickets, or a positive family history) with or without extra-renal symptoms. The study was approved by Padua University Hospital’s Ethical Committee, protocol 0028285 (11 May 2016)

#### 4.1.2. Biopsies

Ten kidney biopsies were collected from patients carrying nine different *CLCN5* mutations (Table 2). All biopsies were performed for diagnostic purposes and available for immunolabeling studies subject to informed consent.

Eight control cortical tissues were obtained from nephrectomies for renal cancer (sites remote from the tumor-bearing renal tissue), disclosing a normal morphology and no immunofluorescence. The study was approved by Padua University Hospital’s Ethical Committee, protocol 0007452 (1 February 2018).

### 4.2. Sanger Sequencing

*CLCN5* gene mutation analysis was performed by Sanger sequencing. Genomic DNA was extracted from peripheral blood using the QIAamp DNA Blood Minikit (Qiagen, Milan, Italy) according to the manufacturer’s instructions. The primers and PCR conditions for amplifying the *CLCN5* gene-coding region and intron–exon boundaries are described elsewhere [82]. The PCR products were analyzed using the Bioanalyzer 2100 (Agilent Technologies, Milan, Italy) and purified with the MinElute PCR Purification Kit (Qiagen, Milan, Italy). Sanger sequencing was done with the BigDye Terminator v1.1 Cycle Sequencing Kit (ThermoFisher Scientific, Milan, Italy) and the ABI-PRISM 3100 Genetic Analyzer (ThermoFisher Scientific, Milan, Italy). The nomenclature of mutations is based on the *CLCN5* cDNA sequence NM_0000844. Missense and splicing mutations were interpreted using the Mutation Taster (MT) [84], and classified according to the American College of Medical Genetics and American College of Pathologists (ACMG/AMP) variant classification guidelines [22]. Mutations were confirmed by sequencing a second independent PCR product.

### 4.3. Whole-Exome Sequencing (WES)

WES was performed at Padua University’s Centro di Ricerca Interdipartimentale per le Biotecnologie Innovative (CRIBI) sequencing center using the Ion Proton System (ThermoFisher Scientific, Milan, Italy), obtaining an average reads coverage of 80X for each sample. Data were analyzed as suggested by the manufacturer, with read alignment using TMAP and variant calling with TSVC, which are both included in the Ion Proton Suite (v 5.0). QueryOR (http://queryor.cribi.unipd.it, accessed on 27 November 2019) [85] was used to analyze and prioritize short-nucleotide variants (SNV). This web-based query platform enables quick, easy, in-depth variant prioritization by aggregating several functional annotations of both genes and variants. The prioritization strategy entails a ranking that sorts results by the number and weight of the criteria met (see below).

Two main approaches were initially used to identify the most promising variants: (1) a gene-centered search, considering known details of genes and associated pathways, and information about the disease and related disorders; and (2) a variant-centered search, focusing on the intrinsic characteristics of variants, such as type (indel, snp, mnp), codon effect (frameshift, missense, and nonsense variants), and genomic position.

In a subsequent prioritization step, variants were ranked by sequencing coverage, minor allele frequency (MAF) values ≤ 0.05, and predicted possible–probable deleteriousness. For this purpose, QueryOR provides coding variant predictions based on several tools, including the well-known SIFT, PolyPhen2, and MT, and the more recent PROVEAN, CADD, and DANN scores [84,86,87,88,89,90]. Sanger sequencing was used to validate variants identified by the in silico prioritization strategy during WES. Table S5 shows the gene names, NCBI reference sequences, primers, and PCR amplification conditions.

Identified variants were checked against relevant database such as Clinvar (https://www.ncbi.nlm.nih.gov/clinvar/, accessed on 27 November 2019) and The Human Gene Mutation Database (HGMD) (http://www.hgmd.cf.ac.uk/ac/index.php, accessed on 27 November 2019), and were classified according to ACMG/AMP guidelines [22].

### 4.4. Immunohistochemistry (IHC)

IHC was conducted on formalin-fixed, paraffin-embedded sections using an indirect immunoperoxidase method. Specimens were treated as previously described [91], and incubated overnight with rabbit anti-human ClC-5 (Sigma-Aldrich, Milan, Italy, cat. HPA000401) diluted 1:200, goat anti-human ClC-3 (Santa Cruz Biotechnologies, Heidelberg, Germany, cat. sc-17572) diluted 1:150, and rabbit anti-human ClC-4 (Sigma-Aldrich, Milan, Italy, cat. HPA063637) diluted 1:50 in PBS at 4 °C in a humidified chamber. A donkey anti-goat IgG-HRP secondary antibody (Santa Cruz Biotechnologies, Heidelberg, Germany, cat. sc-2020) diluted 1:100 was used for ClC-3. Immunolabeling specificity was confirmed by incubating without any primary antibody. Images were acquired with the Diaplan light microscope (Leitz, Como, Italy) and 20X/0.45 objective using a Micropublisher 5.0 RTV camera (Teledyne QImaging, Surrey, BC, Canada).

### 4.5. Immunofluorescence (IF)

IF analyses were performed on serial sections of kidney biopsies. Samples were treated as previously described [92] and incubated overnight with primary antibody (sheep anti-human cubilin [R&D Systems, Minneapolis, MN, USA, cat. AF3700], rabbit anti-human megalin [LS-Bio, Seattle, WA, USA, cat. LS-B105]) diluted 1:100 in PBS 5% BSA at 4 °C. Sections were incubated with the appropriate fluorescent secondary antibody [92]. Nuclei were counterstained with 4′,6-diamidino-2-phenylindole (DAPI). Negative controls were run by omitting the primary antibody. Images were acquired with a DMI6000CS-TCS SP8 fluorescence microscope (Leica Microsystems, Milan, Italy) with a 20X/0.4 objective using a DFC365FX camera (Leica Microsystems, Milan, Italy) and analyzed with the LAS-AF software (Leica Microsystems, Milan, Italy).

### 4.6. Morphometric Analysis

ClC-5, megalin, and cubilin signals on kidney biopsies were quantified by morphometric analysis using Image-Pro Plus 7.0 (Media Cybernetics, Abingdon, United Kingdom). Signals were acquired at 200X with the same time exposure, gain, and intensity for all patients, and quantified excluding the glomerular compartment. For ClC-5, only apical or subapical tubular staining was considered as positive. Quantities were expressed as the mean area covered by pixels (%).

### 4.7. Statistical Analysis

Non-parametric tests (Mann–Whitney U-test) were used due to the small sample size. Results with *p* < 0.05 were considered significant and given as median ± IQR. All analyses were performed with R software version 3.5.1 (R Foundation for Statistical Computing, Vienna, Austria) [93].

## 5. Conclusions

By describing 23 novel *CLCN5* mutations, this study extends the allelic heterogeneity of DD1. Our results on DD1 kidney biopsies provide evidence that ClC-5 is lost in PTCs, and this, in turn, leads to a defective trafficking of megalin and cubilin in these cells.

Using WES to investigate DD3 patients, we did not identify the supposed third Dent disease-causing gene. Instead, our study suggests that likely pathogenic variants in genes encoding components of the endocytic apparatus of tubular cells (megalin, cubilin, NHE3, and NHE-RF3) may have determined DD3 phenotypes. However, except for one patient in whom we identified a known monogenic disease, in the other patients, the presence of variants in more than one gene related in functional networks suggest that we are probably facing oligogenic disorders. Furthermore, our study suggests that DD3 patients are a pool of patients with DD-like phenotypes, which may present atypical phenotypes of known hereditary nephropathies or blended phenotypes.

## Figures and Tables

**Table 1:** Novel mutations in the *CLCN5* gene.

Type of Mutation	Nucleotide	Exon-Intron	Protein	Pathogenicity Assessment	Protein Domain
Frameshift	c.100_101insG	Exon 2	p.(Glu35fs)	Pathogenic (Ib)	A helix, stop in Loop A-B
Frameshift	c.125delA	Exon 3	p.(Glu42fs)	Pathogenic (Ib)	Loop A-B
Frameshift	c.266_267insT	Exon 4	p.(Ile89fs)	Pathogenic (Ib)	B helix, stop in Loop B-C
Frameshift	c.518delT	Exon 6	p.(Ile173fs)	Pathogenic (Ib)	D helix, stop in Loop D-E
Frameshift ^§^	c.691delA	Exon 6	p.(Lys231fs)	Pathogenic (Ia)	Loop F-G, stop at the end of helix G
Frameshift	c.1164_1165insAG	Exon 8	p.(Lys388fs)	Pathogenic (Ib)	L helix, stop in helix M
Frameshift	c.1635_1638delCAAG	Exon 10	p.(Ser545fs)	Pathogenic (Ib)	Q helix, stop in cytoplasmic
Frameshift	c.1657delG	Exon 10	p.(Arg554fs)	Pathogenic (Ib)	Cytoplasmic, stop at the beginning of CBS1 cytoplasmic domain
Frameshift	c.1920delC	Exon 10	p.(Ile641fs)	Pathogenic (Ib)	CBS1 cytoplasmic domain, stop in cytoplasmic
Nonsense	c.1287G>A	Exon 8	p.(Trp429*)	Pathogenic (Ib)	M helix
Nonsense	c.2016C>G	Exon 11	p.(Tyr672*)	Pathogenic (Ib)	Cytoplasmic
Nonsense	c.2128C>T	Exon 11	p.(Gln710*)	Pathogenic (Ib)	Cytoplasmic-beta strand in CBS2 domain
Missense	c.262G>A	Exon 4	p.(Gly88Ser)	Likely pathogenic (IV)	B helix
Missense	c.305G>T	Exon 4	p.(Cys102Phe)	Likely pathogenic (V)	Loop B-C
Missense	c.518T>A	Exon 6	p.(Ile173Lys)	Likely pathogenic (V)	D helix
Missense ^§^	c.608C>G	Exon 6	p.(Ser203Trp)	Pathogenic (II)	E helix
Missense	c.809G>A	Exon 8	p.(Ser270Asn)	Likely pathogenic (IV)	Loop H-I
Missense ^§^	c.922G>A	Exon 8	p.(Val308Met)	Pathogenic (IIIb)	Loop I-J
Missense	c.1565T>A	Exon 10	p.(Val522Asp)	Likely pathogenic (V)	P helix
Missense	c.1619C>T	Exon 10	p.(Ala540Val)	Likely pathogenic (IV)	Q helix
Missense	c.2192A>C	Exon 12	p.(His731Pro)	Likely pathogenic (V)	Cytoplasmic-CBS2 domain
Splicing	c.105+5G>C	Intron 2-splice site	p.?	Likely pathogenic (II)	
Splicing	c.1348-1G>A	Intron 8-splice site	p.?	Pathogenic (Ic)	

^§^*CLCN5* mutations also analyzed in patients’ kidney biopsies.

**Table 2:** Morphometric evaluation of tubular ClC-5 immunolabeling in 10 DD1 patients’ kidney biopsies.

Patient	*CLCN5* Mutation	Age at Biopsy (Years)	Indication for Biopsy	Histopathological Findings	ClC-5 Immunolabeling Morphometric Evaluation (% Positive Area)
1	p.(Thr44fs)	2	Proteinuria	Minimal changes	0.01
2	p.(Lys231fs) (novel)	14	Proteinuria	Normal	0.00
3	p.(Arg34*)	11	Nephrotic syndrome	Chronic interstitial nephritis with global glomerulosclerosis	0.12
4	p.(Arg34*)	6	Proteinuria	Global glomerulosclerosis and IgM nephropathy	0.00
5	p.(Gln600*)	6	Proteinuria	Tubulointerstitial injury with focal glomerulosclerosis	0.05
6	p.(Ser203Trp) (novel)	3	Proteinuria	Normal	0.01
7	p.(Ser261Arg)	4	Heavy proteinuria	Proliferative mesangial glomerulonephritis	0.07
8	p.(Tyr272Cys)	NA	Proteinuria	Normal	0.01
9	p.(Val308Met) (novel)	9	Proteinuria and hematuria	Normal	0.08
10	p.(Trp547Arg)	1	Proteinuria	Normal	0.00

NA: not available.

**Table 3:** Candidate variants for DD3 phenotypes detected by whole-exome sequencing (WES).

Pt ID	Transcript Level Variation	Codon Substitution	Frequency ExAC (European)	Mutation Taster	PROVEAN	DANN	ClinVar	ACMG/AMP Variant Interpretation
***SLC17A1 (NM_005074.3)***								
**AMS**	c.1309G>A	p.(Ala437Thr)	rs1189357572 0.000003 (GnomAD) (0.000008)	Polymorphism (1.000)	Neutral (−1.97)	0.967	NA	VUS
***PDZK1 (NM_002614.4)***								
**AMS**	c.22C>T	p.(Arg8*) homozygous	rs191362962 0.0001157 (0.0001799)	Disease causing automatic	NA	0.998	NA	VUS
***LRP2 (NM_004525.2)***								
**BDA**	c.6727C>T	p.(Arg2243*)	novel	Disease causing automatic (1.000)	NA	0.996	NA	Pathogenic (Ib)
**BDA**	c.242T>A	p.(Ile81Asn)	novel	Disease causing (1.000)	Damaging (−4.62)	0.993	NA	Likely pathogenic (V)
**AMV**	c.6160G>A	p.(Asp2054Asn)	rs138269726 0.0011 (0.0017)	Disease causing (1.000)	Neutral (−1.85)	0.999	Pathogenic allele	Likely pathogenic (II)
**AMT**	c.2006G>A	p.(Gly669Asp)	rs34291900 0.0285 (0.0434)	Disease causing (1.000)	Damaging (−5.44)	0.998	Likely benign allele	Likely benign
**AMT**	c.7894A>G	p.(Asn2632Asp)	rs17848169 0.02951 (0.0426)	Disease causing (1.000)	Neutral (−1.73)	0.452	Likely benign allele	Likely benign
***SLC3A1 (NM_000341.4)***								
**AMS**	c.680G>A	p.(Arg227Gln)	rs142469446 0.0002 (0.0002)	Disease causing (1.000)	Neutral (−1.63)	1	NA	Likely pathogenic (IV)
**AMS**	c.797T>C	p.(Phe266Ser)	rs141587158 0.003 (0.004)	Disease causing (1.000)	Damaging (−4.7)	0.998	NA	Likely pathogenic (IIIb)
***CUBN (NM_001081.3)***								
**AMT**	c.10265C>T	p.(Thr3422Ile)	rs1801230 0.01832 (0.02829)	Disease causing (1.000)	Damaging (−2.94)	0.985	Likely benign allele	Benign
**AMV**	c.7040_7042del	p.(Val2347del)	rs1279549461 (TOPmed) 0.0000001	Disease causing (0.998)	Deleterious (−9.81)	NA	NA	VUS
***SLC9A3 (NM_004174.3)***								
**AMT**	c.848G>A	p.(Arg283His)	rs146899318 0.00033 (0.00036)	Disease causing (0.853)	Damaging (−3.85)	0.999	NA	VUS

Pt: patient, ACMG/AMP: American College of Medical Genetics and American College of Pathologists, NA: not available, VUS: Variant of uncertain significance.

## Supplementary Materials

Supplementary materials can be found at https://www.mdpi.com/1422-0067/21/2/516/s1. Figure S1: ClC-5, ClC-4 and ClC-3 immunolabeling in serial sections of a control kidney. Tubular staining was almost exclusively apical (arrows) for ClC-5, while cytoplasmic staining (arrowheads) was seen for ClC-3, and ClC-5 and much less for ClC-4. Scale bar = 50 μm. Table S1: Clinical phenotypes of 20 patients carrying novel CLCN5 mutations. Table S2: List of phenocopy genes. Table S3: List of the genes prioritized according to scalable kernel-based gene prioritization (SCUBA) [24]. Table S4: DD-like phenotype of patients carrying likely pathogenic variants as detected by whole exome sequencing. Table S5: PCR primer sequences and amplification conditions.
