# Supplementary material for: Genetic Analyses in Dent Disease and Characterization of CLCN5 Mutations in Kidney Biopsies
Source: Int J Mol Sci. 2020 Jan 14;21(2):516. doi: 10.3390/ijms21020516 (PMC7014080; doi:10.3390/ijms21020516)
Supplement: Supplementary file 1 [file ijms-21-00516-s001.pdf]

Supplemental information

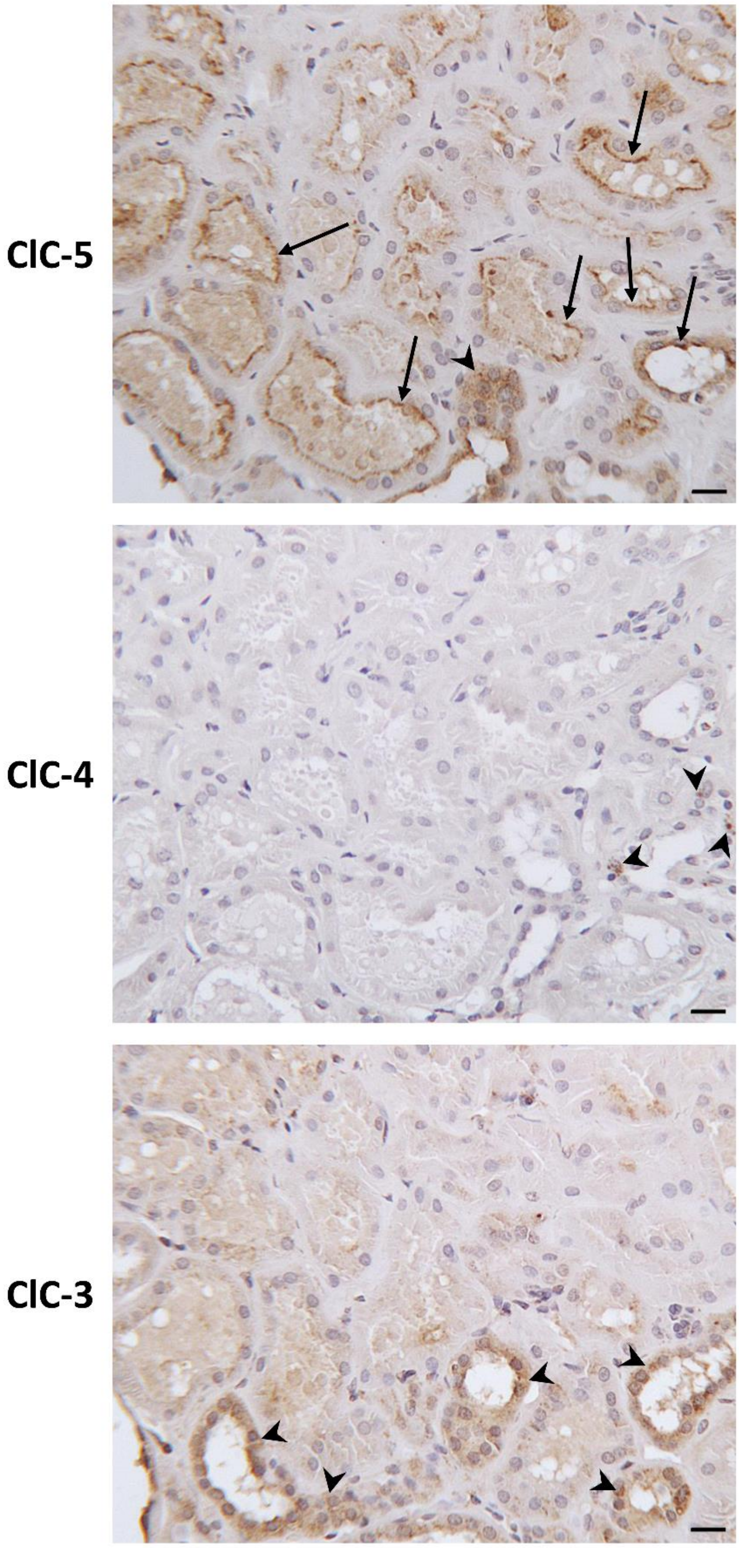

Figure S1. ClC-5, ClC-4 and ClC-3 immunolabeling in serial sections of a control kidney. Tubular staining was almost exclusively apical (arrows) for ClC-5, while cytoplasmic staining (arrowheads) was seen for ClC-3, and ClC-5 and much less for ClC-4. Scale bar = 50μm.

Table S1: Clinical phenotypes of 20 patients carrying novel *CLCN5* mutations

Patient	*CLCN5* mutations	Age at diagnosis (years)	LMWP	Hypercalciuria	Nephrocalcinosis	Nephrolithiasis	Phosphaturic Tubulopathy	Kidney failure	Bone disorders	Extrarenal symptoms
A	p.(Glu35fs)	1	+	-	+	-	-	NA	NA	-
B	p.(Ile89fs)	43	+	-	+	+	+	+	-	-
C	p.(Ile173fs)	9	+	+	-	-	-	NA	NA	-
D	p.(Lys231fs)	8	+	+	+	-	-	NA	-	-
E	p.(Lys388fs)	1	+	+	+	-	-	-	-	-
F	p.(Arg554fs)	2	+	+	+	-	-	-	-	-
G	p.(Ile641fs)	43	+	+	-	-	NA	+	+	-
H	p.(Trp429*)	2	+	NA	+	-	-	NA	+	+
J	p.(Gln710*)	36	+	+	+	+	+	+	NA	+
K	p.(Gly88Ser)	4,5	+	-	NA	NA	-	-	NA	-
I	p.(Cys102Phe)	10	+	NA	-	-	-	NA	NA	-
L	p.(Ile173Lys)	3	+	-	NA	NA	NA	NA	NA	-
M	p.(Ser203Trp)	4	+	-	-	-	-	-	-	-
N	p.(Ser270Asn)	49	+	+	+	-	+	NA	-	-

O	p.(Val308Met)	10	+	+	-	-	-	-	-	-
P	p.(Val522Asp)	3	+	+	-	-	-	+	+	-
Q	p.(Ala540Val)	50	+	+	-	+	-	+	-	+
R	p.(His731Pro)	11	+	+	+	-	NA	-	-	-
S	c.105+5G>C	5	+	NA	NA	+	NA	NA	+	-
T	c.1348-1G>A	4	+	+	-	-	-	-	NA	-
Frequency			20 (100)	12 (72)	9 (55)	4 (21)	3 (18)	5 (38)	4 (28)	3 (14)

NA: not available, +: present, -: absent, Frequencies are shown as number of positive patients for each clinical sign (%)

Extrarenal symptoms were: varus deformities of the lower limbs and dental anomalies (enamel and dentin hypoplasia) (patient H), hyperglobulia and splenomegaly (patient J), and psoriasis (patient Q)

Table S2. List of phenocopy genes

PROXIMAL TUBULOPATHY

AMN (Amnionless)

CA2 (Carbonic Anhydrase 2)

CUBN (Cubilin)

CFTR (CF Transmembrane Conductance Regulator)

CLCN5 (Chloride Voltage-Gated Channel 5)

CTNS (Cystinosin, Lysosomal Cystine Transporter)

DMP1 (Dentin Matrix Acidic Phosphoprotein 1)

ENPP1 (Ectonucleotide Pyrophosphatase/ Phosphodiesterase 1)

FGF23 (Fibroblast Growth Factor 23)

LRP2 (Megalin)

OCRL (OCRL Inositol Polyphosphate-5-Phosphatase)

PHEX (Phosphate Regulating Endopeptidase Homolog X-Linked)

SLC1A1 (Solute Carrier Family 1 Member 1)

SLC22A12 (Solute Carrier Family 22 Member 12)

SLC2A2 (Solute Carrier Family 2 Member 2)

SLC34A1 (Solute Carrier Family 34 Member 1)

SLC34A3 (Solute Carrier Family 34 Member 3)

SLC3A1 (Solute Carrier Family 3 Member 1)

SLC4A4 (Solute Carrier Family 4 Member 4)

SLC5A2 (Solute Carrier Family 5 Member 2)

SLC7A7(Solute Carrier Family 7 Member 7)

SLC7A9 (Solute Carrier Family 7 Member 9)

DISTAL TUBULOPATHY

AQP2 (Aquaporin 2)

ATP6V0A4 (ATPase H+ Transporting V0 Subunit A4)

ATP6V1B1 (ATPase H+ Transporting V1 Subunit B1)

AVPR2 (Arginine Vasopressin Receptor 2

BSND (Barttin CLCNK Type Accessory Beta Subunit)

CASR (Calcium Sensing Receptor)

CLCNKA (Chloride Voltage-Gated Channel Ka)

CLCNKB (Chloride Voltage-Gated Channel Kb)

CLDN16 (Claudin 16)

CLDN19 (Claudin 19)

CNNM2 (Cyclin And CBS Domain Divalent Metal Cation Transport Mediator 2)

EGF (Epidermal Growth Factor)

FXYD2 (FXYD Domain Containing Ion Transport Regulator 2)

GNA11 (G Protein Subunit Alpha 11)

AP2S1 (Adaptor Related Protein Complex 2 Subunit Sigma 1)

HNF1B (HNF1 Homeobox B)

KCNA1 (Potassium Voltage-Gated Channel Subfamily A Member 1*)*

KCNJ1 (Potassium Inwardly Rectifying Channel Subfamily J Member 1)

KCNJ10 (Potassium Inwardly Rectifying Channel Subfamily J Member 10)

MUC1 (Mucin 1, Cell Surface Associated)

REN (Renin)

SLC12A1 (Solute Carrier Family 12 Member 1)

SLC12A3 (Solute Carrier Family 12 Member 3)

SLC41A3 (Solute Carrier Family 41 Member 3)

SLC4A1 (Solute Carrier Family 4 Member 1 - Diego Blood Group)

TRPM6 (Transient Receptor Potential Cation Channel Subfamily M Member 6)

UMOD (Uromodulin)

NEPHROLITHIASIS

AGXT (Alanine--Glyoxylate And Serine--Pyruvate Aminotransferase)

APRT (Adenine Phosphoribosyltransferase)

CYP24A1 (Cytochrome P450 Family 24 Subfamily A Member 1)

GRHPR (Glyoxylate And Hydroxypyruvate Reductase)

HOGA1 (4-Hydroxy-2-Oxoglutarate Aldolase 1)

XDH (Xanthine Dehydrogenase)

PROXIMAL TUBULAR ENDOCYTIC PATHWAY

ARAP3 (ArfGAP With RhoGAP Domain, Ankyrin Repeat And PH Domain 3)

CHMP4A (Charged Multivesicular Body Protein 4A)

DAB2 (Disabled Homolog 2)

PDZK1 (PDZ Domain Containing 1)

SLC9A3 (Solute Carrier Family 9 Member A3)

SLC9A3R1 (SLC9A3 Regulator 1)

SLC9A3R2 (SLC9A3 Regulator 2)

Table S3. List of the genes prioritized according to scalable kernel-based gene prioritization (SCUBA) [24]

Rank	Gene	ID	Score	Status	Haploinsufficiency Score	Gene Intolerance Score
1	*OCRL*	4952	1	SeedGene	0.289	0.6401273885
2	*CLCN5*	1184	0.8467	SeedGene	0.457	0.4455060156
3	*RAB6A*	5870	0.09665	Predicted	0	0.387237556
4	*RAB14*	51552	0.09665	Predicted	0.112	0.471455532
5	*RAB1A*	5861	0.09665	Predicted	0.626	0.2819061099
6	*RAB8A*	4218	0.09665	Predicted	0.174	0.6457891012
7	*RAB5A*	5868	0.08744	Predicted	0.726	0.6854210899
8	*CFL1*	1072	0.07404	Predicted	0.312	0.6032083038
9	*GRB2*	2885	0.06225	Predicted	1,000	0.5546119368
10	*CLTC*	1213	0.04975	Predicted	0.590	0.95152158528
11	*CLCNKB*	1188	0.04203	Predicted	0.119	0.1469686247
12	*CLCN2*	1181	0.04203	Predicted	0.159	0.2034088228
13	*CLCN1*	1180	0.04203	Predicted	0.356	0.3247817882
14	*CLCN6*	1185	0.04203	Predicted	0.381	0.96827081859
15	*WWP1*	11059	0.04076	Predicted	0.702	0.8487850908
16	*CDC42*	998	0.04033	Predicted	0	0.5379806558
17	*RAC1*	5879	0.04033	Predicted	0.991	0.5875206417
18	*GATA2*	2624	0.02837	Predicted	0.315	0.7443972635
19	*ARHGEF9*	23229	0.02575	Predicted	0.209	0.5510733664
20	*ATP6V1C1*	528	0.02268	Predicted	0.252	0.7879806558
21	*ATP6V0A2*	23545	0.02268	Predicted	0.226	0.8252535975
22	*ATP6V1F*	9296	0.02268	Predicted	0.090	0.4505189903
23	*ATP6V0C*	527	0.02268	Predicted	0.136	0.5712432177

24	*ATP6V0D1*	9114	0.02268	Predicted	0.145	0.713670677
25	*ATP6V0B*	533	0.02268	Predicted	0.323	0.5061335221
26	*ATP6V1G3*	127124	0.02268	Predicted	0.080	0.0806794055000001
27	*ATP6AP1*	537	0.02268	Predicted	0.065	0.2230478887
28	*ATP6V1B2*	526	0.02268	Predicted	0.835	0.7566643076
29	*ATP6V1E2*	90423	0.02268	Predicted	0.121	0.3968506723
30	*ATP6V1H*	51606	0.02268	Predicted	0.112	0.8598136353
31	*ATP6V0D2*	245972	0.02268	Predicted	0.223	0.3664189667
32	*ATP6V1B1*	525	0.02268	Predicted	0.137	0.7724699222
33	*ATP6V1A*	523	0.02268	Predicted	0.211	0.7485255957
34	*ATP6V1E1*	529	0.02268	Predicted	0.168	0.437485256
35	*ATP6V1G2*	534	0.02268	Predicted	0.109	0.471455532
36	*ATP6V0A1*	535	0.02268	Predicted	0.418	0.8479594244
37	*ATP6V0E2*	155066	0.02268	Predicted	0	0
38	*ATP6V0A4*	50617	0.02268	Predicted	0.219	0.5932413305
39	*TCIRG1*	10312	0.02268	Predicted	0.204	0.93424156641
40	*ATP6V1G1*	9550	0.02268	Predicted	0.126	0.2332507667
41	*ATP6V1C2*	245973	0.02268	Predicted	0.111	0.4790634584
42	*ATP6V1D*	51382	0.02268	Predicted	0.279	0.4859636707
43	*ATP6V0E1*	8992	0.02268	Predicted	0.142	0.437485256
44	*E2F4*	1874	0.02222	Predicted	0.877	0.8606393017
45	*MAGI1*	9223	0.02115	Predicted	0	0.95641660769
46	*WWP2*	11060	0.02115	Predicted	0.753	0.7820240623
47	*TRPC7*	57113	0.01961	Predicted	0.287	0.5602146733
48	*UBC*	7316	0.01961	Predicted	0	0.8776244397

49	*UBB*	7314	0.01961	Predicted	0.911	0.5875206417
50	*NEDD4L*	23327	0.01961	Predicted	0.378	0.94214437367
51	*ATP2A3*	489	0.01961	Predicted	0.111	0.96260910592
52	*TRPC6*	7225	0.01961	Predicted	0.141	0.94916253833
53	*RPS27A*	6233	0.01961	Predicted	0.271	0.387237556
54	*ATP2A1*	487	0.01961	Predicted	0.698	0.95730125029
55	*RAF1*	5894	0.01961	Predicted	0.999	0.8355744279
56	*UBA52*	7311	0.01961	Predicted	0	0.4896201934
57	*ATP2A2*	488	0.01961	Predicted	0.255	0.96962727058
58	*TRPC3*	7222	0.01961	Predicted	0.294	0.94585987261
59	*PDZD11*	51248	0.01776	Predicted	0.081	0.3904222694
60	*HK3*	3101	0.01696	Predicted	0.805	0.8044939844
61	*NUP85*	79902	0.01696	Predicted	0.365	0.90386883699
62	*NUP133*	55746	0.01696	Predicted	0.554	0.1964496343
63	*G6PC*	2538	0.01696	Predicted	0.844	0.8606393017
64	*FTL*	2512	0.01696	Predicted	0.078	0.5875206417
65	*HK1*	3098	0.01696	Predicted	0.816	0.94361877801
66	*SEH1L*	81929	0.01696	Predicted	0.779	0.5487143194
67	*NUP160*	23279	0.01696	Predicted	0.847	0.97676338759
68	*CFTR*	1080	0.01696	Predicted	0.687	0.7826728002
69	*SLC33A1*	9197	0.01696	Predicted	0.567	0.3280844539
70	*NUP37*	79023	0.01696	Predicted	0.940	0.6993394669
71	*GCK*	2645	0.01696	Predicted	0.830	0.7106628922
72	*FTH1*	2495	0.01696	Predicted	0	0.387237556
73	*G6PC2*	57818	0.01696	Predicted	0.110	0.1482071243

74	*TUSC3*	7991	0.01696	Predicted	0.544	0.6962727058
75	*NUP107*	57122	0.01696	Predicted	0.905	0.8013682472
76	*TFRC*	7037	0.01696	Predicted	0.352	0.85326728
77	*NUP98*	4928	0.01696	Predicted	0.932	0.94987025242
78	*HK2*	3099	0.01696	Predicted	0.546	0.90622788393
79	*NUP43*	348995	0.01696	Predicted	0.475	0.7231068648
80	*RANBP2*	5903	0.01696	Predicted	0.934	0.9666784619
81	*G6PC3*	92579	0.01696	Predicted	0.157	0.3187072423
82	*MYO9B*	4650	0.01535	Predicted	0.155	0.8907171503
83	*PRKCG*	5582	0.01535	Predicted	0.336	0.8550365652
84	*PRKCB*	5579	0.01535	Predicted	0	0.8776244397
85	*A2M*	2	0.01535	Predicted	0.264	0.3924274593
86	*ABR*	29	0.01535	Predicted	0.502	0.92197452229
87	*OPHN1*	4983	0.01535	Predicted	0.126	0.1979830149
88	*CDIPT*	10423	0.01535	Predicted	0.116	0.6457891012
89	*PIK3CB*	5291	0.01535	Predicted	0.996	0.95895258316
90	*MTMR14*	64419	0.01535	Predicted	0.282	0.90038924275
91	*RHOH*	399	0.01535	Predicted	0.246	0.6602972399
92	*ARAP1*	116985	0.01535	Predicted	0	0.96066289219
93	*ARHGAP15*	55843	0.01535	Predicted	0.783	0.8283793347
94	*TAGAP*	117289	0.01535	Predicted	0.568	0.1013800425
95	*MTMR6*	9107	0.01535	Predicted	0.254	0.0991389479
96	*ITSN1*	6453	0.01535	Predicted	0.646	0.9827789573
97	*SYDE2*	84144	0.01535	Predicted	0.351	0.1417197452
98	*ARHGAP25*	9938	0.01535	Predicted	0.735	0.1317527719

99	*TPTE2*	93492	0.01535	Predicted	0.074	0.5955414013
100	*KALRN*	8997	0.01535	Predicted	0.627	0.99728709601

Table S4: DD-like phenotype of patients carrying likely pathogenic variants as detected by whole exome sequencing

	**BDA**	**AMT**	**AMV**	**AMS**
Age at diagnosis (years)	68	26	6	38
LMWP	+	+	+	-
Proteinuria	+	+	+	+
Hypercalciuria	+	+	+	+
Nephrocalcinosis	NA	NA	+	-
Nephrolithiasis	+	+	-	+
Phosphaturic Tubulopathy	+	+	-	+
Kidney failure	+	NA	-	NA
Aminoaciduria	-	NA	-	NA
Familiarity	+	+	-	+
Bone disorders	+	NA	-	-
Extrarenal symptoms	+	+	-	-

NA: not available; +: present, -: absent

Extrarenal symptoms in BDA: hearing loss and eye blindness, osteomalacia. This case was described in reference [63].

Extrarenal symptoms in AMT: short stature

Table S5: PCR primer sequences and amplification conditions

Gene Name	NCBI Reference Sequence	Primer name	Primer (5'-3')	[Primer] µM	Size (bp)	Cycles	T°a (°C)	[$MgCl_2$] mM
SLC17A1	NM_005074.3	SLC17A1_Ex12_For	ACCTTGAAAAGATGCAGAAAACT	0.4	551	30	60 (td 65)	1.5
		SLC17A1_Ex12_Rev	CCAAACCTGCACCCGTTATT	0.4				
SLC3A1	NG_008233.1	SLC3A1_Ex3_For	TGCCTGGCCTGTCATATGTT	0.4	253	30	60 (td 65)	1.5
		SLC3A1_Ex3_Rev	CAGTTGTTGGGTGGAATGGT	0.4				
		SLC3A1_Ex4_For	GGATCAGGGAGGGCAATGAT	0.4	235	30	60 (td 65)	1.5
		SLC3A1_Ex4_Rev	CTTGAACATCAGGATTGCGGA	0.4				
PDZK1	NM_002614.4	PDZK1_Ex3_For	AGCAAGTTCTGGAAGGTGTC	0.4	296	35	60	1.5
		PDZK1_Ex3_Rev	TGCTGGGCTACACTTCTCAA	0.4				
LRP2	NG_012634.1	LRP_Ex3_For	TAATGGGGAGGATGATTGCCA	0.4	248	30	60 (td 65)	3
		LRP_Ex3_Rev	GTAACTTTCTTTGCGACAGGT	0.4				
		LRP_Ex15_For	TGCAATTTAACCCTGTGCAT	0.4	203	30	60 (td 65)	3
		LRP_Ex15_Rev	TGCCAACAACGTCCAAAACA	0.4				
		LRP_Ex37_For	CAACATGAATGCCTGTCAGC	0.4	278	40	60	1
		LRP_Ex37_Rev	TCATGGCACCAGGGATTTCA	0.4				
		LRP_Ex39_For	TCGGGCTGACTATGGGCAGA	0.4	213	30	60 (td 65)	3
		LRP_Ex39_Rev	CGACTGCCATAACGAATCAC	0.4				
		LRP_Ex42_For	GTGGATCGTGAAGTCATTGTCA	0.4	235	30	60 (td 65)	3
		LRP_Ex42_Rev	CCCCATTAAACTGTTCACAAGGA	0.4				

CUBN	NG_008967.1	CUBN_Ex46_For	CTTTCTTGCTTGCTTGCTTTCT	0.4	250	35	64	1
		CUBN_Ex46_Rev	TGTCCCAGATCTCCACGAAG	0.4				
		CUBN_Ex64_For	TCCTGTGAAGTTGAAAGAGCAC	0.4	224	30	62	1
		CUBN_Ex64_Rev	CTGAGTTCTCGATGCCAAGTG	0.4				
SLC9A3	NG_046804.1	SLC9A3_Ex5_For	GTCCTCTACCCAGTGTCCTT	0.4	246	35	60	1.5
		SLC9A3_Ex5_Rev	TGGAGAAGCTCGGGAGGA	0.4				

td: touch-down profile, bp: base pair
